# The Actual Efficacy of an Air Purifier at Different Outdoor PM_2.5_ Concentrations in Residential Houses with Different Airtightness

**DOI:** 10.3390/toxics10100616

**Published:** 2022-10-17

**Authors:** Dongho Shin, Younghun Kim, Keejung Hong, Gunhee Lee, Inyong Park, Bangwoo Han

**Affiliations:** 1 Department of Sustainable Environment Research, Korea Institute of Machinery & Materials, Daejeon 34103, Korea; 2 Department of Mechanical Engineering, Yonsei University, Seoul 03722, Korea

**Keywords:** clean air delivery rate, air purifier, residential house, ACH50, indoor

## Abstract

It is important to control airborne particles in residential houses for protecting human health. Indoor particulate matter of <2.5 μm (PM_2.5_) can be effectively monitored and managed using an air purifier. In this study, the actual clean air delivery rates in residential houses (CADR_Actual_) were acquired by comparing decay rates of fine particles with and without operations of the air purifier under actual conditions, following the standard CADR of an air purifier obtained in a closed test chamber. The measurements of CADR_Actual_ at different outdoor PM_2.5_ concentrations over a month in two residential houses revealed different airtightness levels, compared to the standardized clean air delivery rate of the air purifier (CADR_AP_). Air changes per hour at 50 Pa (ACH50) was 4.8 h^−1^ for “house A” (built in 2007) and 2.1 h^−1^ for “house B” (built in 2018). The CADR of the air purifier used in this study was 10.6 m^3^/min, while the averaged CADR_Actual_ at the “house A” was 7.2 m^3^/min (approximately 66% of the CADR of the air purifier) and 9.5 m^3^/min at “house B” (approximately 90% of the CADR of the air purifier). Under the outdoor PM_2.5_ concentrations of <35 μg/m^3^, the averaged CADR_Actual_ of house A and house B were 7.8 ± 0.3 and 9.7 ± 0.4 m^3^/min, respectively. However, under the outdoor PM_2.5_ concentrations of >35 μg/m^3^, the analogous averaged concentrations were 6.8 ± 0.6 and 9.6 ± 0.3 m^3^/min for houses A and B, respectively. The measured CADR_Actual_ agreed well with the theoretical estimates of CADR_Actual_ acquired by the mass balance equation using the infiltration rate of ACH50/20. We also estimated CADR_Actual_/CADR_AP_ for house C built in 2017, where the ACH50 was 1.8 h^−1^. Overall, this study demonstrated how CADR_Actual_/CADR_AP_ of an air purifier at residential houses can be predicted according to outdoor PM_2.5_ concentration and airtightness of the house. As shown, it can be closer to 1 at lower ACH50 houses and at lower outdoor PM_2.5_ concentrations.

## 1. Introduction

South Korea suffers from poor air quality, driven by high atmospheric concentrations of PM_2.5_ and PM_10_, either emitted from coal-fired power plants and automobiles [1,2,3] or transported by seasonal yellow dust inflow from China [4,5]. Fine particles are designated as a group 1 carcinogen by the World Health Organization (WHO) because long exposure to such particles can cause respiratory and cardiovascular diseases in the human body [6,7,8]. Globally, most people spend indoors >90% of the day [9,10,11], being exposed to deteriorated air quality because the average concentration of fine particles indoors is approximately 6 times higher than outdoors [12]. Sources of PM indoors include the inflow of fine particles from outside [13,14], cooking [15], indoor smoking [16], and incense [17]. Most of the particles generated from these sources are fine and ultra-fine particles that can cause lung disease by penetrating the alveoli when inhaled by humans [18]. Alarmingly, annual premature deaths driven by the indoor air pollution are arguably comparable to those caused by outdoor ambient air pollution [19].

The deterioration of outdoor air quality is accompanied by visible changes in the atmospheric environment because when PM_2.5_ levels are high, the atmosphere becomes visibly hazier. Thus, we can approximately estimate the conditions of outdoor air quality and take action accordingly by wearing a face mask or reducing outdoor exertion for reducing personal exposure to fine particles. However, it is not easy to recognize indoor PM_2.5_ levels due to the short light scattering path in indoor environments. Although awareness of people about indoor air quality is increasing, which prompts the popularity of residential products such as air purifiers, air ventilation units, and kitchen range hoods; However, most people use such products without the knowledge of using them effectively [20].

An air purifier is an electric device to reduce fine particles and gaseous species in indoor air. Numerous studies have been focused on effectively removing fine and ultrafine particles using air purifiers with high efficiency particle filters [21,22] and electrostatic precipitators [23,24]. Moreover, many other studies aimed to expand the functions of air purifiers by removing gaseous contaminants or biological particles with photocatalysts [25,26], non-thermal plasma [27,28] etc. Further, some studies evaluated the performance of air purifiers for various particles generated indoors. It was investigated on allergens from pets such as cats and dogs [29,30], tobacco smoke particles [31,32], and particles from cooking [33,34,35]. However, most previous studies related to air purifiers were limited to strictly defined laboratory environments. To date, only a few field studies have evaluated the efficacy of air cleaners affected by the inflow of automobile exhaust particles and outdoor particles in urban environments [36,37,38,39]. However, the external influence on air purifiers in residential houses is still poorly understood.

In this context, the performance of an air purifier can be evaluated based on the clean air delivery rate (CADR) that is normally used in the standard test protocols (AHAM-AC-1-2020, SPS-KACA002-132, GB/T 18801-2015) in a completely enclosed test chamber. However, in residential houses, it is strongly affected by external factors such as outdoor PM_2.5_ concentrations, wind speed, and airtightness of the house. The efficacy of the air purifier in residential houses varies due to some external factors, thereby which is prompting quantitative analysis of these effects on the air purifier. In this light, the spatial variance of the performance of an air purifier was recently investigated in an actual in-use office room and revealed that CADR in the office room was considerably lower than the one determined by the standard chamber [40]. It was simply interpreted that the degradation was attributable to the different size distributions of the realistic and the standard test particles despite such external factors as outdoor PM_2.5_ levels and the airtightness of the office potentially affected their results strongly. 

In this study, the CADRs of an air purifier in actual residential houses with different airtightness have been investigated at different PM_2.5_ concentrations. To this end, (1) they were compared to that, acquired in the standard chamber using the same test aerosols; and (2) a theoretical prediction tool for actual CADRs was established at different outdoor PM_2.5_ concentrations and airtightness of the houses.

## 2. Methods

**Figure 1:**
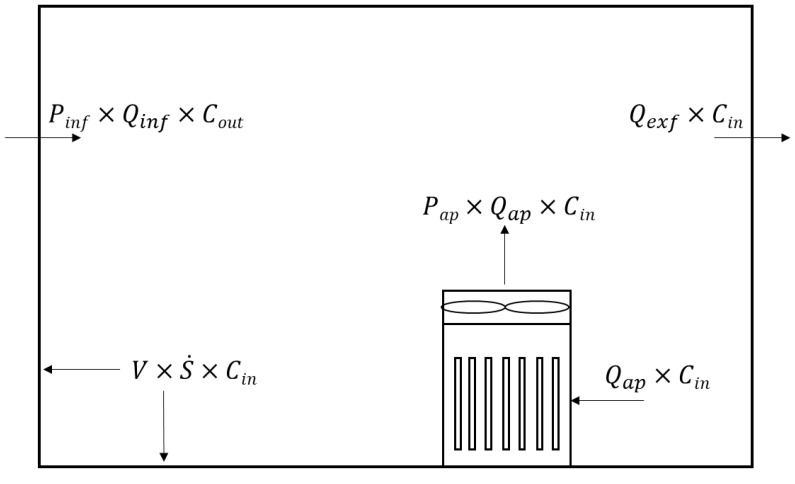
Schematic diagram of indoor particle dynamic process with an air purifier in a space.

Figure 1 illustrates a schematic diagram modeling factors affecting indoor particle concentration in a residential house using an air purifier. The factors of modeling are penetration (P_AP_) and flow rates (Q_AP_) of an air purifier, particle deposition rate ($\dot{S}$) indoors, flow rate of infiltration (Q_inf_) and exfiltration (Q_exf_); penetration of infiltration (P_inf_) and outdoor (C_out_) and indoor (C_in_) particle concentration. The mass conservation equation is formalized based on the modelling:

(1) $$V\frac{{\mathrm{dC}}_{\mathrm{in}}}{\mathrm{dt}}=\varepsilon_{\mathrm{AP}}\times \left(P_{\mathrm{AP}}\times Q_{\mathrm{AP}}\times C_{\mathrm{in}}-Q_{\mathrm{AP}}\times C_{\mathrm{in}}\right)+P_{\inf}\times Q_{\inf}\times C_{\mathrm{out}}-Q_{\mathrm{exf}}\times C_{\mathrm{in}}-V\times \dot{S}\times C_{\mathrm{in}}$$

The mass conservation equation was established for the change in the mass concentration of particles generated and removed indoors. To this end, four mechanisms were organized: removal by an air purifier, infiltration of outdoor particle concentration, exfiltration of indoor particle concentration and removal by natural deposition. In Equation (1), C_out_ is the outdoor particle concentration (μg/m^3^), C_in_ is the indoor particle concentration (μg/m^3^). Furthermore, ε_AP_ is the dimensionless value of indoor air circulation by the air purifier. According to Noh and Oh [41], when using a stand-alone type air purifier of 10 m^3^/min in a space of approximately 50 m^3^, the ε_AP_ is 0.81, which was used in this study. P_AP_ is the penetration of particles in the air purifier filter. For the P_AP_ used in the calculation, 0.03 was used as the particle collection efficiency of the air purifier filter was 97%. Q_AP_ represents the volume flow rate of the air purifier (m^3^/min). The air flow rates of the air purifier are 11 m^3^/min. P_inf_ is the penetration of particles flowing indoors from outside. For P_inf_, 0.9 was used when the differential pressure between indoors and outdoors was 4 Pa in the general atmospheric condition based on a particle size of 0.3 μm [42,43]. Q_inf_ is the flow rate of infiltration (m^3^/min), Q_exf_ is the flow rate of exfiltration (m^3^/min), $\dot{S}$ is the natural deposition rate (min^−1^), and V is the volume (m^3^). Equation (1) can be summarized as follows:

(2) $${\mathrm{CADR}}_{\mathrm{AP}}=\varepsilon_{\mathrm{AP}}\times \left(1-P_{\mathrm{AP}}\right)\times Q_{\mathrm{AP}}$$

(3) $$V\frac{{\mathrm{dC}}_{\mathrm{in}}}{\mathrm{dt}}=-\left({\mathrm{CADR}}_{\mathrm{AP}}+Q_{\mathrm{exf}}+V\times \dot{S}\right)\times C_{\mathrm{in}}+P_{\inf}\times Q_{\inf}\times C_{\mathrm{out}}$$

Clean air delivery rate of an air purifier (CADR_AP_) was defined in Equation (2) above. CADR_AP_ is fundamentally equal to the product of air flow rate, collection efficiency of an air purifier filter, and air circulation rate by the air purifier. Equation (1) was used to summarize the formalization in Equation (3). The change of the indoor concentration over time was determined by the indoor factors such as air purifiers, natural deposition, and air flowing into and out of the test houses through which external particle flows into the test houses. The transient solution of the mass balance equation was established as:

(4) $$C_{\mathrm{in}}\left(t\right)=C_{\mathrm{in},0}\times \exp\left[-\frac{{\mathrm{CADR}}_{\mathrm{AP}}+Q_{\mathrm{exf}}+V\times \dot{S}}{V}t\right]+\frac{{\;P}_{\inf}\times Q_{\inf}\times C_{\mathrm{out}}}{{\mathrm{CADR}}_{\mathrm{AP}}+Q_{\mathrm{exf}}+V\times \dot{S}}\times \left[1-\exp\left[-\frac{{\mathrm{CADR}}_{\mathrm{AP}}+Q_{\mathrm{exf}}+V\times \dot{S}}{V}t\right]\right]$$

By using Equation (4), the changes in indoor concentration when the air purifier was operated was theoretically calculated. In the calculation, the outdoor particle concentration and house airtightness in residential houses were considered. The result was then compared with the actual measured values in the test houses.

The Association of Home Appliance Manufacturers (AHAM) [44] defines CADR as the reduction by an air purifier in a closed chamber methodologically. Theoretically, the regression of particle concentration follows a first-order decay model:

(5) $$C_{\mathrm{in}}\left(t\right)=C_{\mathrm{in},0}\exp\left[-\mathrm{kt}\right]$$

where

C_in_(t) = concentration at time *t*
C_in,0_(t) = initial concentration at t = 0
k = decay constant
t = time (min)

The time-resolved decay constant k is calculated statistically using a linear regression of ln C(t_i_) and t_i_ with the following formula:

(6) $$k=\left[\frac{S_{xy}}{S_{xx}}\right]$$

where,

$$S_{\mathrm{xy}}=\sum_{i=1}^{n}t_{i}\ln C_{t_{i}}-\frac{1}{n}\left(\sum_{i=1}^{n}t_{i}\right)\left(\sum_{i=1}^{n}\ln C_{t_{i}}\right),\;S_{\mathrm{xx}}=\sum_{i=1}^{n}{\left(t_{i}\right)}^{2}-\frac{1}{n}{\left(\sum_{i=1}^{n}t_{i}\right)}^{2}$$

Using Equations (5) and (6), the AHAM method for calculating CADR is

(7) $$\mathrm{CADR}=V\;\left(k_{\mathrm{on}}-k_{\mathrm{off}}\right)$$

where

CADR = clean air delivery rate (m^3^/min)
V = volume of test chamber (m^3^)
k_on_ = total decay rate (min^−1^)
k_off_ = natural decay rate (min^−1^)

In this study, test air purifier CADR (CADR_AP_) was measured using an air purifier cleaning ability test in a standard test chamber measuring 30 m^3^. After generating a 1% solution of potassium chloride (KCl) at the concentration of approximately 3 × 10^8^ particles/m^3^ through an atomizer (3076, TSI, USA), the natural decay rate, k_off_ of fine particles in the test chamber was measured for 30 min by using Equations (5) and (7).

For comparing the standardized CADR from the test chamber, we defined the actual clean air delivery rates in residential houses under actual environments as follows.

(8) $${\mathrm{CADR}}_{\mathrm{Actual}}=V\times {\left.\left(\frac{{\mathrm{lnC}}_{2}-{\mathrm{lnC}}_{1}}{t_{2}-t_{1}}\right)\right|}_{\mathrm{on}}-V\times {\left.\left(\frac{{\mathrm{lnC}}_{2}-{\mathrm{lnC}}_{1}}{t_{2}-t_{1}}\right)\right|}_{\mathrm{off}}={\;\mathrm{CADR}}_{\mathrm{Actual},\;\mathrm{on}}-{\mathrm{CADR}}_{\mathrm{Actual},\;\mathrm{off}}$$

In practice, CADR_Actual_ represents the rate of decrease with time of particle concentration measured indoors. Here, C_1_ and C_2_ represent the concentrations indoors according to time and *t* is time (min). CADR_Actual,on_ is the decrease in indoor concentration over time when the air purifier is turned on and CADR_Actual,off_ is when the air purifier is turned off, and CADR_Actual_ was defined by CADR_Actual,on_ minus CADR_Actual,off_. CADR_Actual_ reflects the change in indoor concentration caused by an air purifier. 

In Equation (4), besides the time-dependent exponential term, a term $\frac{{\;P}_{\inf}\times Q_{\inf}\times C_{\mathrm{out}}}{{\mathrm{CADR}}_{\mathrm{AP}}+Q_{\mathrm{exf}}+V\times \dot{S}}$ includes the outdoor particle concentration (C_out_). This implies that the indoor particle concentration changes with time and is affected by the outdoor particle concentration. Thus, it does not take the form of an exponential function. CADR_AP_, reflecting the performance of an air purifier represents a function of the decreased rate of indoor particle concentration over time. However, due to the outdoor particle concentration term, the indoor particle concentration does not decrease exponentially with time. Therefore, this study introduced the concept of CADR_Actual_. In this way, the effect of an air purifier on indoor particle concentration was evaluated, while only considering the change in concentration over time. In this study, CADR_Actual_ was calculated with t_1_ being 0 min and t_2_ being 20 min. 

The experiment was conducted using standard test protocols (SPS-KACA002-132) with the ventilation fan and bathroom fan closed, and all the windows and doors closed. Before supplying the test particles, an air purifier for cleaning was used. The cleaning ensured that the concentration of particles sized 0.3–1.0 μm in the room was ≤~2 × 10^7^ particles/m^3^. The particles were generated in this state, while the stirring fan was simultaneously operated to achieve particle agitation. When the concentration of the 0.3–1.0 μm particles reached approximately 3–5 × 10^8^ particles/m^3^ which is the indoor concentration mentioned in the standard protocol, particle generation was stopped and the stirring fan was stopped after 5 min of operation to completely agitate the particles. Furthermore, CADR_Actual,off_ was calculated from the concentration measured for 20 min. After that, the particle concentration was again generated to 3–5 × 10^8^ particles/m^3^. Then, the test air purifier was operated and the degree of decrease in the particle concentration was quantified to calculate the CADR_Actual,on_. Using these values and Equation (8), the CADR_Actual_ was calculated. 

## 3. Experimental Setup

**Figure 2:**
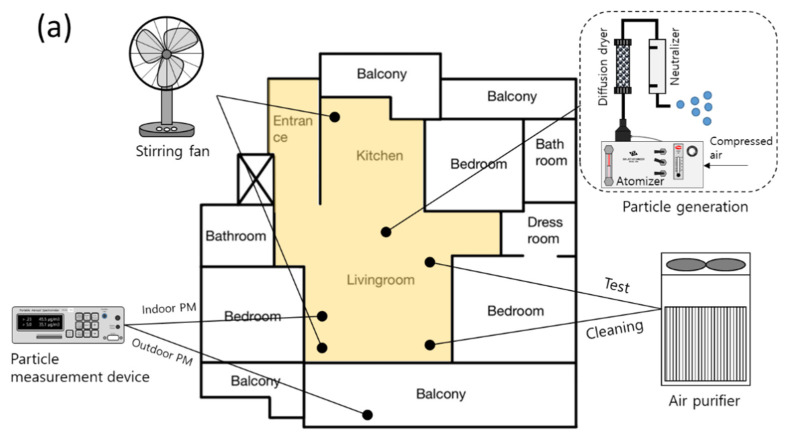

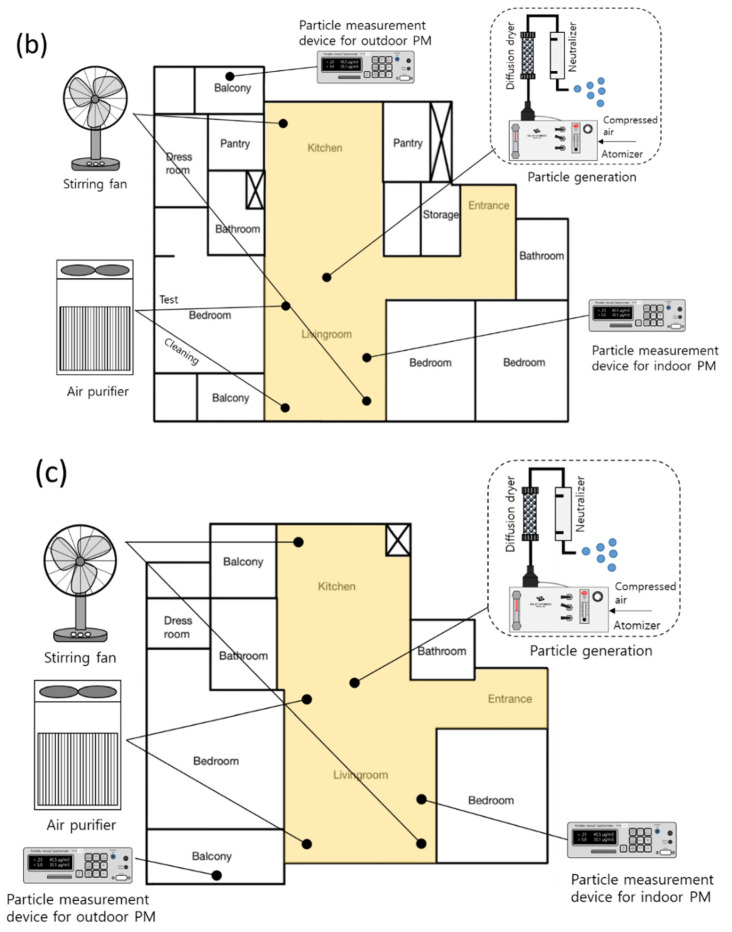
Floor plan of three test houses ((**a**) A house, (**b**) B house, and (**c**) C house) and the layout of experimental devices. A yellow colored space of a living room with an entrance and a kitchen is confined as a test volume in this study.

Figure 2a–c illustrates a drawing of the residential house used in the experiment. Table 1 summarizes the information of each house. Figure 2a is an apartment (house A) built in 2007 located in Daejeon (South Korea). House A has a dedicated area of 84.9 m^2^, where the living and kitchen area used for the experiment is 37.4 m^2^ with an experimental volume of 86 m^3^. Measurements were carried out during January, and it was winter.

Figure 2b shows an apartment (house B) completed in 2018 located in Sejong City (South Korea). house B is a balcony-extended apartment with an exclusive area of 72.5 m^2^, the living and kitchen area of 36.7 m^2^ and the volume of 84.5 m^3^. Although the exclusive area is smaller than that of house A, the area of the living room and kitchen are similar. Measurements were carried out during March, and it was spring.

Figure 2c shows an apartment (house C) completed in 2017 located in Sejong City (South Korea). Its exclusive area is 50.3 m^2^, the living and kitchen area used for the experiment is 34.6 m^2^ and the volume is 79 m^3^. It has a simple structure, good air circulation and like house B, it has a balcony extension structure. Although the exclusive area is small, the living and kitchen area is 79 m^3^, which makes it different from houses A and B. Measurements were carried out during a week of May, and it was spring.

**Figure 3:**
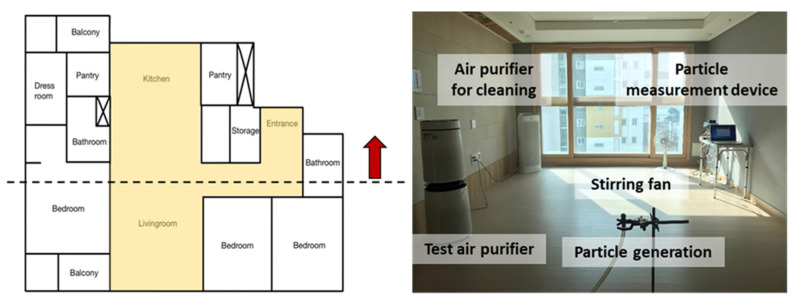
A photo of the set experimental devices at the test house.

Measurements were performed using the same experimental equipment in all test houses; Figure 3 shows a photo of the experimental set-up. A particle generator was placed in the middle of the living room and kitchen, where the particles were generated using a six-jet atomizer (9306, TSI, Shoreview, MN, USA) with the 1% KCl solution. The generated particles passed through a diffusion dryer and a neutralizer to generate only completely neutralized KCl particles. The indoor particle concentration was measured by placing the optical particle counter (OPC 1.109, Grimm, Ainring, Germany) on the inner wall of the living room, which did not look at the air purifier. The outdoor particle concentration was measured by OPC at the balcony. OPCs for indoor and outdoor measurements were calibrated and tested and compared through simultaneous measurement. OPC is a measurement device that uses the light scattering method. As for the concentration, one piece of data was obtained every 6 s, and in this experiment, the data were organized as 1-min averages. The test air purifier was placed at the wall opposite the OPC where it is usually placed at home. In this setup, a sufficient distance from the OPC was placed to prevent inaccurate concentration measurements. The test air purifier is cylindrical, and has an air inlet from the side, and an air outlet to the top. The air flow rate of the air purifier is 11 m^3^/min and the collection efficiency of the air purifier filter is 97%. CADR_AP_ of the test air purifier was 10.6 m^3^/min and CADR_AP_ for each level of the test air purifier was 1.68, 4.37, 6.02, and 10.60 m^3^/min. The stirring fans were placed at both ends of the living room and the kitchen to ensure that the particle concentration could be evenly distributed throughout the house when particles were generated. 

The data used in each experiment were arranged based on data on sunny days except for snowy or rainy days to reduce the effect on weather conditions. In addition, the experiment was conducted only during the daytime. Room temperature was maintained at 24 ± 2 °C and relative humidity was maintained at 50–60% at all test houses. In the case of house A, where the experiment was performed in winter, the difference in indoor and outdoor temperature was larger than that of houses B and C, which were tested in spring, but it was confirmed that the CADR_Actual_ based on the difference in the indoor and outdoor temperatures did not have a significant effect as a result of arranging.

## 4. Results

**Figure 4:**
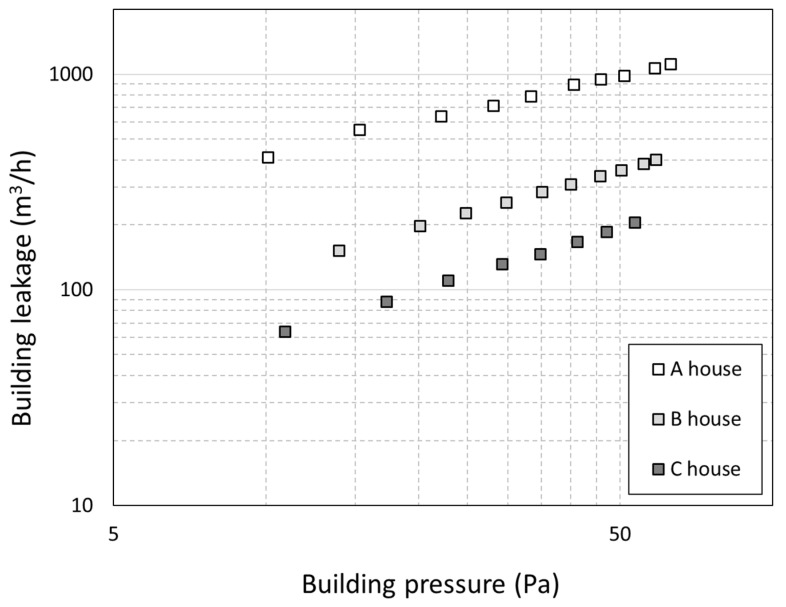
Air leakage flow rate in houses according to indoor envelope pressure for three test houses.

The airtightness at house A and house B was measured according to the EN 13829 [45] and ASTM E779-10 [46] standards. Figure 4 shows the averaged measured estimate of the airtightness measured by pressurization and decompression. The airtightness of the house was determined by measuring the amount of air required to supply or bleed air to the test space to maintain the corresponding pressure from 13 to 60 Pa. Note that airtightness is hereafter expressed as ACH50 (ACH50 is the abbreviation for air changes per hour at 50 Pa pressure differential). In this study, Q_inf_ and Q_exf_ were defined as the functions of airtightness. As the experiment was performed under normal atmospheric pressure, ACH was defined as follows according to [47] as shown in Equations (9) and (10):

(9) $$\mathrm{ACH}=\frac{\mathrm{ACH}50}{20}$$

(10) $$Q_{\inf}=Q_{\mathrm{exf}}=V\times \frac{\mathrm{ACH}}{60}$$

where, ACH is the number of times that the total air volume in a room or space is completely removed and replaced in an hour under atmospheric pressure. It should be noted that some previous studies discussed how to define ACH based on airtightness. In this study, the method defined by the Kronvall–Persily model was used for the analysis [47]. 

The airtightness test was conducted in the same way in houses A, B, and C. The kitchen hood, kitchen sink drain, ventilation diffuser, toilet drain, and ventilation fan were all blocked, and a fan for an airtightness test was installed on the front door to perform pressurization and decompression. ACH50 of houses A, B, and C was 4.8, 2.3, and 1.7 h^−1^, indicating that house A had the worst airtightness.

**Figure 5:**
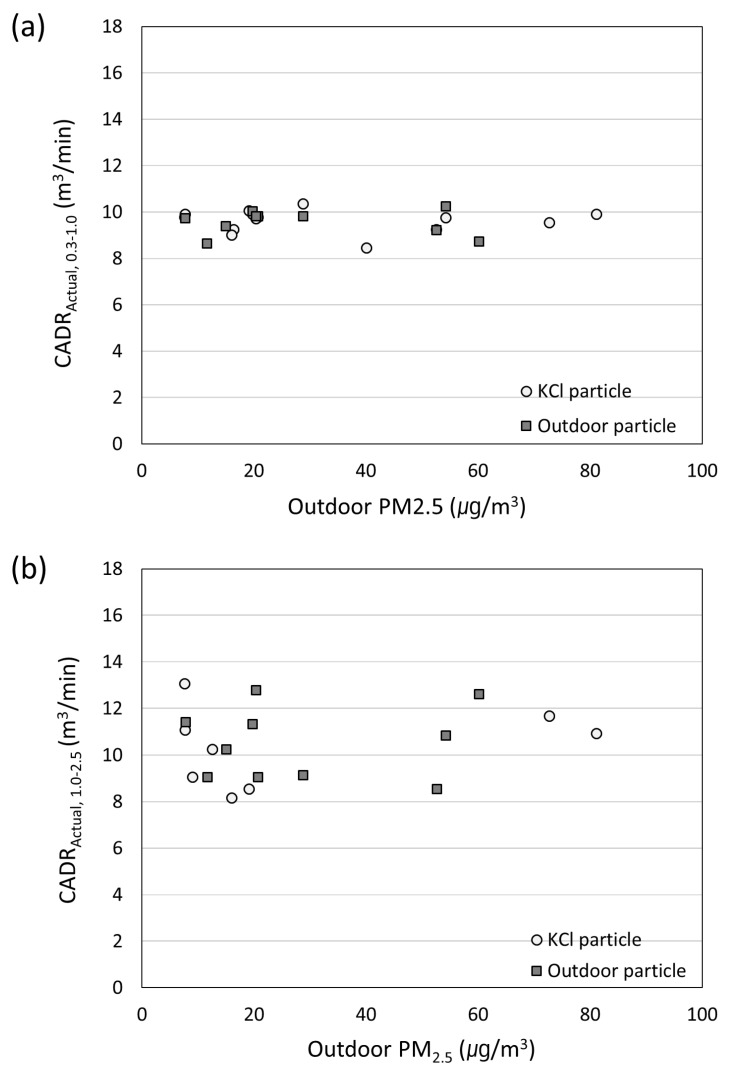
CADR_Actual_ of an air purifier measured at a test house using the KCl test particles and outdoor atmospheric particles for (**a**) 0.3–1.0 μm particles and (**b**) 1.0–2.5 μm particles.

Figure 5 shows CADR_Actual_ of 0.3–1.0 μm (a) and 1.0–2.5 μm (b). The experiment was performed using KCl particles and atmospheric particles in the house B. When experimenting with KCl particles, all windows were closed and KCl particles were sprayed to calculate CADR_Actual_. When the experiment was performed using the atmospheric particles, the indoor PM concentration and the outdoor PM concentration were matched. After that, the window was closed and the air purifier was operated to calculate CADR_Actual_. The reason for the comparison by classifying them into 0.3–1.0 μm and 1.0–2.5 μm is that the particle distribution of the atmospheric particles appeared as a bimodal distribution back and forth (1 μm). 

Here, 10 measurements were performed using KCl particles and atmospheric particles to verify whether the KCl particles can be used for evaluating the performance of the air purifier in house B. The average CADR_Actual_ depending on the outdoor PM_2.5_ was found to be close to 9.62 m^3^/min for KCl particles and 9.47 m^3^/min for atmospheric particles. The corresponding standard deviations were found to be 0.49 and 0.51, thus indicating appropriate values. The average CADR_Actual_ within the 1.0–2.5 μm range, shown in Figure 5b, corresponded to 10.34 and 10.47 m^3^/min, respectively. In this case, the corresponding standard deviations were 1.68 and 1.62, respectively, thereby manifesting the values above 0.3–1.0 μm. In theory, the larger the particle size, the higher the particle collection efficiency due to inertia in the air purifier filter, and the shorter its residence time in the air. Thus, the average was likely higher than CADR_Actual_ of 0.3–1.0 μm. These experimental results confirmed that one can evaluate the air purifier in the test house through KCl particles. In this way, it is possible to further evaluate the same performance as that of the atmospheric dust. On this basis, the experiment was performed using KCl particles for controlling parameters and for constantly matching the generated particles.

**Figure 6:**
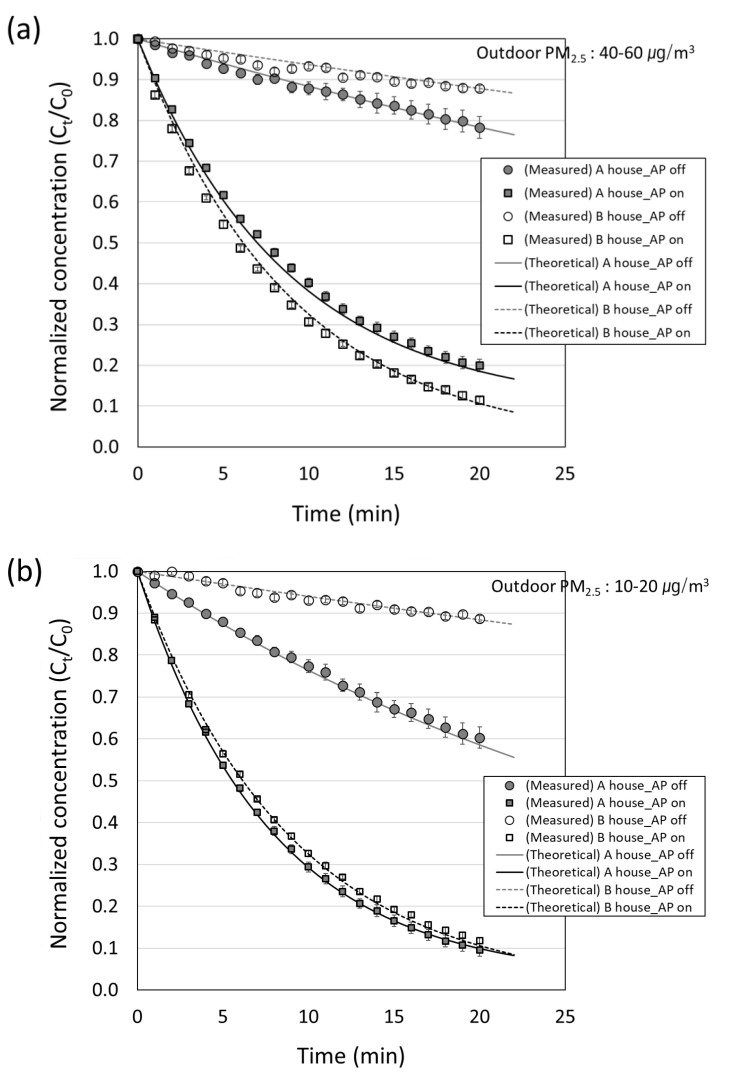
Normalized number concentration of 0.3–1.0 μm particles with operations of an air purifier on and off at two test houses according to time. Outdoor PM2.5 concentrations are in the range of (**a**) 40–60 μg/m^3^ and (**b**) 10–20 μg/m^3^.

Figure 6a shows the change in indoor concentration (C_in_), compared to the initial concentration of 0.3–1.0 μm particles over time when the outdoor PM_2.5_ in house A and house B was 40–60 μg/m^3^, thus manifesting the poor outdoor air quality. In this figure, the circle reflects the measured value for the air purifier turned off, and the square reflects the measured value for the air purifier turned on. Of them, the inner colored area of the circle indicates house A; otherwise house B is implied. The solid line reflects the theoretical calculation value of house A and the dotted line reflects house B using Equation (4). The analysis showed that for house A, ACH50 was 4.8 h^−1^, thereby indicating that the leakage rate of house A was 14.7 m^3^/min at 50 Pa. According to Equations (9) and (10), infiltration and exfiltration flow rate (Q_inf_ and Q_exf_) of house A under normal atmospheric pressure was 0.74 m^3^/min, while for house B, ACH50 was 2.1 h^−1^. At this time, the infiltration and exfiltration flow rate (Q_inf_, Q_exf_) was 0.15 m^3^/min. The theoretical calculation assumes that the flow rates for the infiltration and the exfiltration were the same. In this study, 0.05 h^−1^ was applied as the deposition rate for 0.3–1.0 μm particles [48]. In the air purifier off condition, the normalized concentration reduction in house A was larger than in house B. However, in the air purifier on condition, it was lower than in house B. The airtightness was poor and the amount of inflowing and outflowing outdoor air of house A was higher than that of house B. Therefore, on the one hand, the concentration reduction in house A was strong. On the other hand, when the air purifier was operated, the decrease in concentration over time in house B was stronger compared to that in house A. This difference was driven by the high outdoor concentration of PM_2.5_, which implied that the degree of external fine particle inflow was high in house A. In turn, the decrease in the indoor concentration was low. Figure 6b shows the change in indoor concentration (C_in_), compared to the initial concentration of 0.3–1.0 μm particles over time when the outdoor PM_2.5_ in house A and house B was 10–20 μg/m^3^, (e.g., good air quality). The comparison of the air purifier “off” condition in house A, on the day of high outdoor PM_2.5_ (Figure 6a) demonstrated that the indoor concentration decreased by approximately 20%, compared to the initial concentration after 20 min. On the day of low outdoor PM_2.5_ (Figure 6b), the decrease of approximately 40% was identified. The decrease was driven by bad airtightness and the concentration was rapidly decreased due to the inflow of air with a lower concentration than the initial concentration from the outside. In house B, the airtightness was good, thereby implying that a decrease in the concentration after 20 min was similar to approximately 10% regardless of the outdoor PM_2.5_. Moreover, it was confirmed that it was less affected by the outdoor PM_2.5_ than the house A. Figure 5a,b confirm that the indoor concentration after 20 min under the operating conditions of the air purifier in house B was 10% of the initial level, which decreased regardless of the outdoor PM_2.5_. For house A, the indoor concentration after 20 min was found to be 20% of the initial concentration when the outdoor PM_2.5_ was high. Moreover, when the outdoor PM_2.5_ was low, the indoor concentration was 10% of the initial concentration. The analysis confirmed that such a concentration was affected by the outdoor PM_2.5_. It is therefore reasonable to suggest that the indoor cleaning effect of the air purifier was more influenced by the outdoor PM_2.5_ in houses with poor airtight performance. Moreover, it was found that the calculated value from Equation (4) and the measured value agreed well.

**Figure 7:**
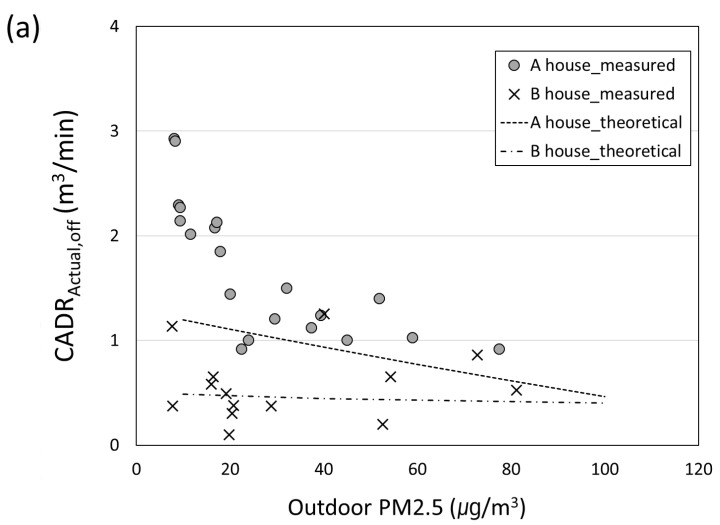

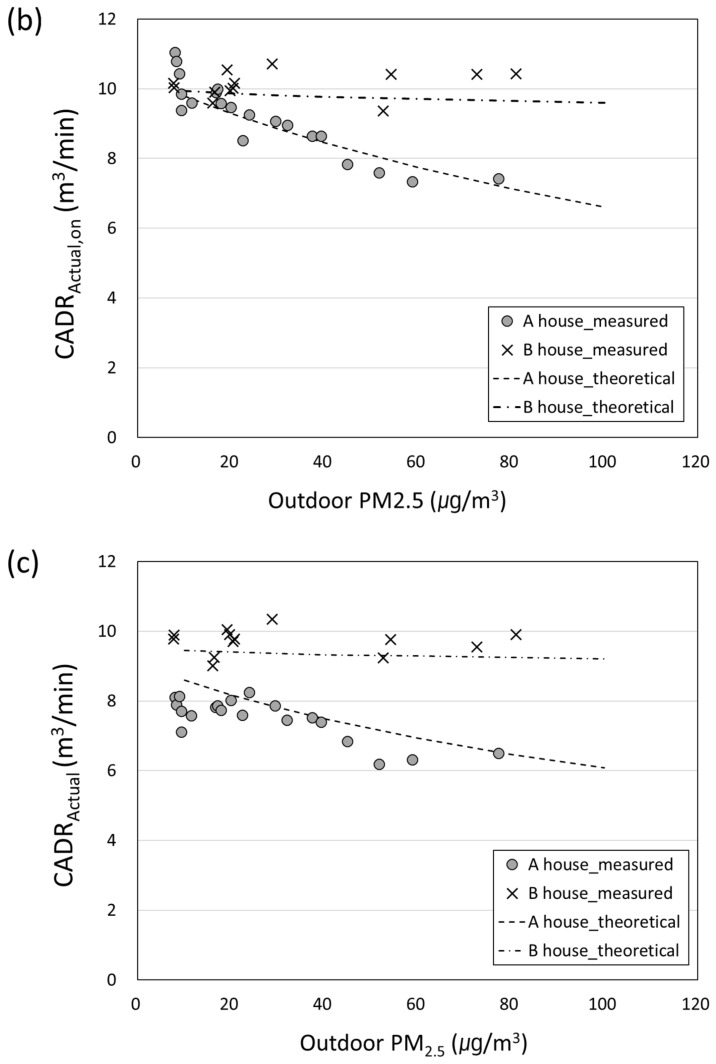
Experimental and theoretical CADR_Actual_ of an air purifier for 0.3–1.0 μm particles at different outdoor PM_2.5_ concentrations. (**a**) is acquired with an air purifier off (CADR_Actual,off_) (**b**) is with an air purifier on (CADR_Actual,on_)and (**c**) is CADR_Actual_ (=CADR_Actual,on_ − CADR_Actual,off_).

Figure 7 shows the (a) CADR_Actual,off_, (b) CADR_Actual,on_, and (c) CADR_Actual_ according to the outdoor PM_2.5_ in house A and house B. The test of an air purifier effect in a residential house was performed 20 times in house A and 13 times in house B on 0.3–1.0 μm particles. Then, the results were compared with the CADR_Actual_ values obtained from Equations (5) and (8). Figure 7a shows that the theoretical and measured values were similar when the outdoor PM_2.5_ was 20 μg/m^3^ or higher in house A. However, the measured value was significantly higher than the theoretical value when the outdoor PM_2.5_ was 20 µg/m^3^ or less. This pattern was driven by the high wind speed outdoors on a day when the outdoor PM_2.5_ was low, which exacerbated the amount of air flowing into the test house from the outdoors. As a result, a high CADR_Actual,off_ was identified. Moreover, wind speed measurements demonstrated that the wind speed was 1.2 m/s for the concentration of >20 μg/m^3^, while the average outside wind speed was 2.5 m/s below 20 μg/m^3^, thereby manifesting a twofold difference. At the same time, house B exhibited low ACH50 of 2.1 h^−1^, thereby confirming that CADR_Actual,off_ was maintained as low as 0.5 m^3^/min on average even with the changes in outdoor PM_2.5_. Figure 7b illustrates the comparison of the theoretical and measured values for indoor CADR_Actual,on_ according to the outdoor PM_2.5_ when the air purifier was operating. For house A, CADR_Actual,on_ decreased down to 7.4 m^3^/min at 75 μg/m^3^ or higher, thereby signifying adverse levels of PM_2.5_. At the same time, on the days when the outdoor PM_2.5_ was <10 μg/m^3^, the CADR_Actual,on_ could mount to 11 m^3^/min, thereby exceeding the CADR level of the air purifier. Moreover, house B maintained approximately 10 m^3^/min regardless of outdoor PM_2.5_. Figure 7c shows the results of CADR_Actual_ according to the outdoor PM_2.5_ as measured and theoretical values. As seen, the measured CADR_Actual_ of house A was maintained at 7.7 m^3^/min, while the outside air PM_2.5_ was below the normal level of 35 μg/m^3^. Moreover, it decreased gradually down to approximately 6.3 m^3^/min above 35 μg/m^3^. Thus, it can be theoretically confirmed that CADR_Actual_ decreased as the concentration of PM_2.5_ increased under the influence of the outdoor PM_2.5_. However, for house B, it was hardly affected by the outdoor PM_2.5_, and CADR_Actual_ was maintained at approximately 9.5 m^3^/min. For house A with poor airtightness, only 73% of CADR_AP_ was effective on the days when the outdoor PM_2.5_ was low. Moreover, 60% of CADR_AP_ was effective on the days when the outdoor PM_2.5_ was high. However, for house B with good airtightness, 90% of CADR_AP_ was identified regardless of the outdoor PM_2.5_. 

**Figure 8:**
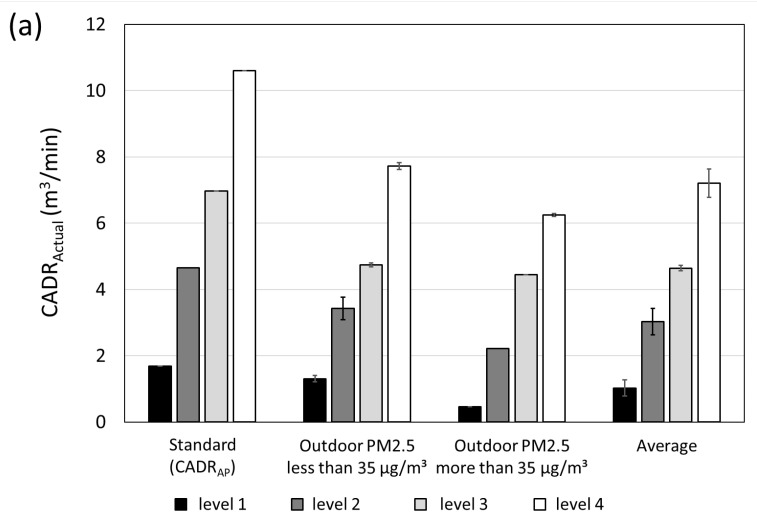

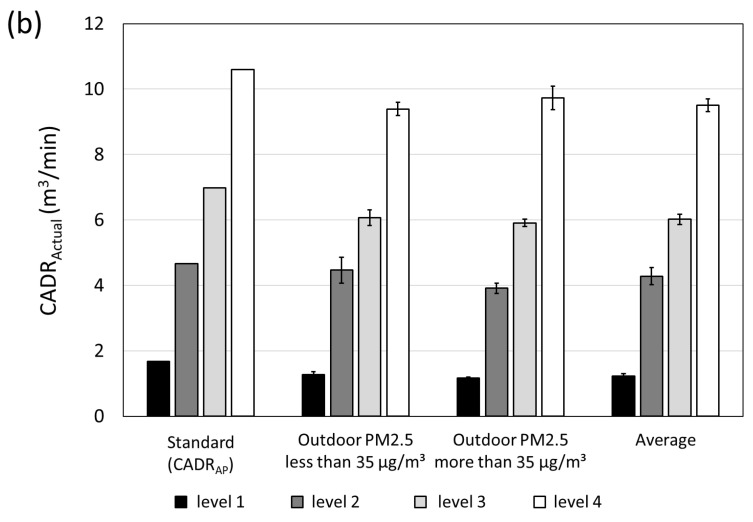
Comparison of CADR_Actual_ to CADR_AP_ (standard CADR) at (**a**) house A and (**b**) house B according to different outdoor PM_2.5_ level ranges.

Figure 8 shows the comparison of CADR_Actual_ for each flow rate level of an air purifier with the CADR_AP_ measured by the air purifier test standard in the test chamber for 0.3–1.0 μm particles in house A (a) and house B (b). The classification was performed based on the level of outdoor PM_2.5_ of 35 μg/m^3^ above which PM_2.5_ can be deemed to be adverse. Figure 8a demonstrates that when the outdoor PM_2.5_ was <35 μg/m^3^, CADR_Actual_ estimates were 78, 74, 68, and 73% of CADR_AP_ for each level, thus exhibiting the average performance of 73%. At the outdoor PM_2.5_ of ≥35 μg/m^3^ these estimates were 27, 48, 64, and 59% for each stage, respectively, thereby signifying the average performance of 50%. As seen, the lower air flow rate levels, the lower the CADR_Actual_ compared to the CADR_AP_. This pattern was due to the following phenomenon: smaller the flow rates of the air purifier, the longer it takes to purify the air in the indoor space, and the more affected by the outdoor PM_2.5_ during that time. Overall, this finding indicates that the effect of the air purifier would be further weakened when the applied space is larger than the application space of an air purifier. Figure 8b shows that when the outdoor air PM_2.5_ was ≤35 μg/m^3^, CADR_Actual_ estimates were 76, 96, 87, and 89%, compared to the standard for each level, respectively, thus exhibiting the average performance of 87%. However, when the outdoor PM_2.5_ was ≥35 μg/m^3^ these estimates were 70, 84, 85, and 92%, thus marking the average performance of 83%. Unlike other levels, the CADR_Actual_ of the first level was found within the 70% range. This is rather low, compared to the standard because the flow rate of the first level is not sufficient to purify the living space of the test house. When the air purifier was actually used in a house, it could not exhibit the anticipated expected effect. Due to this, it was possible to confirm the effect of outdoor PM_2.5_ according to the airtightness of the house. 

**Figure 9:**
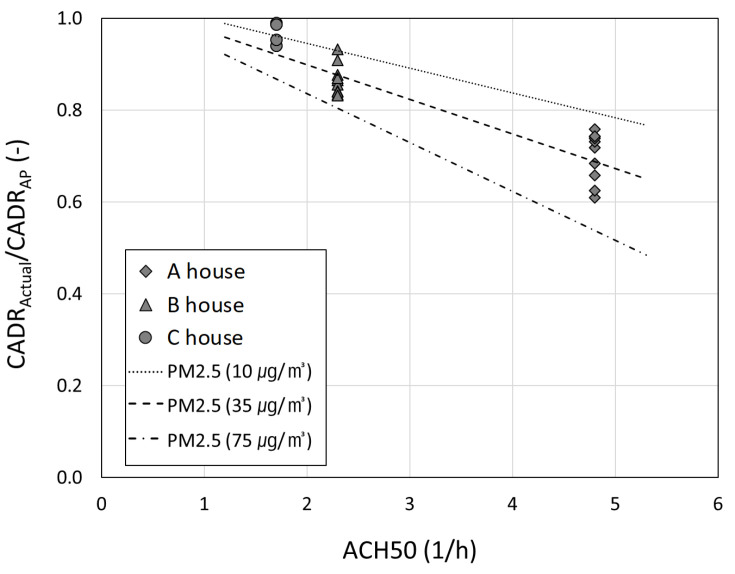
Comparison of experimental and theoretical CADR_Actual_ to standard CADR_AP_ at different PM_2.5_ concentrations according to ACH50 of houses.

Figure 9 shows the CADR_Actual_/CADR_AP_ at different outdoor PM_2.5_ concentrations according to the ACH50 of residential houses. As mentioned, the theoretical CADR_Actual_/CADR_AP_ according to the ACH50 calculated from Equation (5) at the outdoor PM_2.5_ concentrations of 10, 35, 75 μg/m^3^ (see the dotted lines in Figure 9). Here, the CADR_Actual_/CADR_AP_ were acquired not only in houses A and B, but also in house C, thereby exhibiting fairly good airtightness with the ACH50 of 1.8 h^−1^. By comparing the experimental results in house C with different airtightness through Figure 9, it was confirmed that CADR_actual_ varies according to airtightness. It was also demonstrated that the smaller the ACH50 (the higher the airtightness) of residential houses can bolster the air purifier’s cleaning efficacy and the nearer its standard efficacy. 

Overall, on the one hand, the efficacy of an air purifier in a house with an ACH50 of 4.8 h^−1^ can reach only 75% of the original standard, when the outdoor PM_2.5_ is 25 μg/m^3^ (the annual average of PM_2.5_ in South Korea). On the other hand, it can reach about 90% of the original standard in a house with an ACH50 of 2.1 h^−1^ and approximately 95% in a house with an ACH50 of 1.8 h^−1^ at the PM_2.5_ level of 25 μg/m^3^.

## 5. Conclusions

This study analyzed the performance of an air purifier in residential houses according to outdoor PM_2.5_ and the airtightness of the houses. It was found that the actual efficacy of the analyzed air purifier in a poor airtightness house with ACH50 of 4.8 h^−1^ was only approximately 60% of the original standard, previously indicated by a standard protocol. However, up to approximately 90% can be achieved in a house with ACH50 of 1.8 h^−1^ at the outdoor PM_2.5_ levels of >35 μg/m^3^. The actual performance of the air purifier in the residential houses for outdoor PM_2.5_ concentrations and the airtightness of the houses were also theoretically estimated by a mass balance model. It was shown that the theoretical estimates were markedly consistent with those of the experiments. Overall, the higher airtightness of the houses can lead to a higher efficacy of an air purifier, as indicated by the experiment in residential houses. These findings indicate that it is necessary to apply an air purifier with a much higher CADR value in an actual house with relatively weak airtightness to acquire the same decay rate as the standard CADR in a closed chamber. In addition, when using the air purifier at home, it will give a guideline to effectively manage the indoor air quality by using the air purifier at different outdoor PM_2.5_ and airtightness of the house. It will also provide a useful evaluation protocol for the performance of an air purifier in actual environmental conditions.

## Figures and Tables

**Table 1:** Summarized experimental factors by test houses.

Site	Exclusive Area (m^2^)	Experimental Area (m^2^)	Experimental Volume (m^3^)	Built Year	Deposition Rate (1/h)	ACH50 (1/h)
30 m^3^ chamber	30			-	0.05	0.00
House A	84.9	37.4	86	2007	0.05	4.8
House B	72.5	36.7	84.5	2018	0.05	2.3
House C	50.3	34.6	79	2017	0.05	1.7

## Data Availability

Not applicable.
